# DMT analogues: *N*-ethyl-*N*-propyl­tryptamine and *N*-allyl-*N*-methytryptamine as their hydro­fumarate salts

**DOI:** 10.1107/S2056989020008683

**Published:** 2020-07-03

**Authors:** Andrew R. Chadeayne, Duyen N. K. Pham, James A. Golen, David R. Manke

**Affiliations:** a CaaMTech, LLC, 58 East Sunset Way, Suite 209, Issaquah, WA 98027, USA; b University of Massachusetts Dartmouth, 285 Old Westport Road, North Dartmouth, MA 02747, USA

**Keywords:** crystal structure, indoles, tryptamines, hydrogen bonds

## Abstract

The solid-state structures of the hydro­fumarate salts of two *N*,*N*-di­alkyl­tryptamines, namely *N*-ethyl-*N*-propyl­tryptammonium (EPT) hydro­fumarate {systematic name: [2-(1*H*-indol-3-yl)eth­yl](meth­yl)propyl­aza­nium 3-carb­oxy­prop-2-enoate}, C_15_H_23_N_2_^+^·C_4_H_3_O_4_^−^, and *N*-allyl-*N*-methyl­tryptammonium (MALT) hydro­fumarate {systematic name: [2-(1*H*-indol-3-yl)eth­yl](meth­yl)(prop-2-en-1-yl)aza­nium 3-carb­oxy­prop-2-enoate}, C_14_H_19_N_2_^+^·C_4_H_3_O_4_^−^, are reported. Both compounds possess a protonated tryptammonium cation, and a hydro­fumarate anion in the asymmetric unit. The ethyl group of the EPT cation is modeled as a two-component disorder with 50% occupancy for each component. In the extended structure, N—H⋯O and O—H⋯O hydrogen bonds generate infinite two-dimensional networks parallel to the (001) plane for both compounds.

## Abstract 2

The structures of the hydro­fumarate salts of two *N*,*N*-di­methyl­tryptamine (DMT) derivatives, the synthetic psychedelics *N*-ethyl-*N*-propyl­tryptamine (EPT) and *N*-allyl-*N*-methyl­tryptamine (MALT), are reported.

## Chemical context   

Ayahuasca is the traditional spiritual medicine of the indigenous people of the Amazon basin, and has a history of use in religious ceremonies dating back to the 1400′s or earlier. It is an herbal tea that is made by boiling a mixture of leaves and bark. The leaves of the *Psychotria viridis* plant contain about 0.3% of *N*,*N*-di­methyl­tryptamine (DMT) by mass, which is the primary psychoactive in ayahuasca. The bark of the *Banisteriopsis caapi* vine contains many different β-carbolines; these β-carbolines function as mono­amine oxidase (MAO) inhibitors, which prevent the degradation of DMT in the human gut. Without the inhibition of mono­amine oxidase, DMT is not orally active (Cameron & Olson, 2018 ▸).

In a report earlier this year, β-carboline MAO inhibitors were identified in species of ‘magic mushrooms’, where the primary psychedelic, psilocin, can be similarly degraded by MAO. This is the first instance of a synchronous biosynthesis of an active ingredient and the inhibitor of its degradation in a natural psychedelic species (Blei *et al.*, 2020 ▸). Psilocin (4-hy­droxy-*N*,*N*-di­methyl­tryptamine) is orally active in the absence of MAO inhibitors, indicating that the 4-hy­droxy substitution makes the compound more resistant to deamination by MAO (Sherwood *et al.*, 2020 ▸). The presence of β-carbolines in ‘magic mushrooms’ and the varied activity of psilocin and DMT bring many questions forward on the nature of cooperative activity among chemicals in psychotropic natural products.

This class of traditional psychedelics, as well as synthetic variants, have started to gain a great deal of inter­est as anti­depressants and anxiolytics (Johnson *et al.*, 2019 ▸; Jiménez-Garrido *et al.*, 2020 ▸). Given the renewed inter­est in using psychedelic tryptamines as therapeutics, there is growing urgency to perform fundamental physical and biological characterization on these compounds for the benefit of downstream research. This is particularly true in the examination of structure–activity relationships between compounds and also the examination of their cooperative biological activity. A better understanding of these areas would facilitate the research and development of formulations tailored for specific ailments. Two synthetic analogues of DMT are *N*-ethyl-*N*-propyl­tryptamine (EPT) and *N*-methyl-*N*-allyl­tryptamine (MALT), both of which have very limited reports in literature (Ascic *et al.*, 2012 ▸; Brandt *et al.*, 2005*a* ▸,*b* ▸). The preparation of pure crystalline forms of these compounds is essential to conducting meaningful biological studies and ultimately developing drug products. Herein, we report the first solid-state structural characterization of EPT and MALT as their hydro­fumarate salts, (I) and (II), including the first reported salt of MALT.
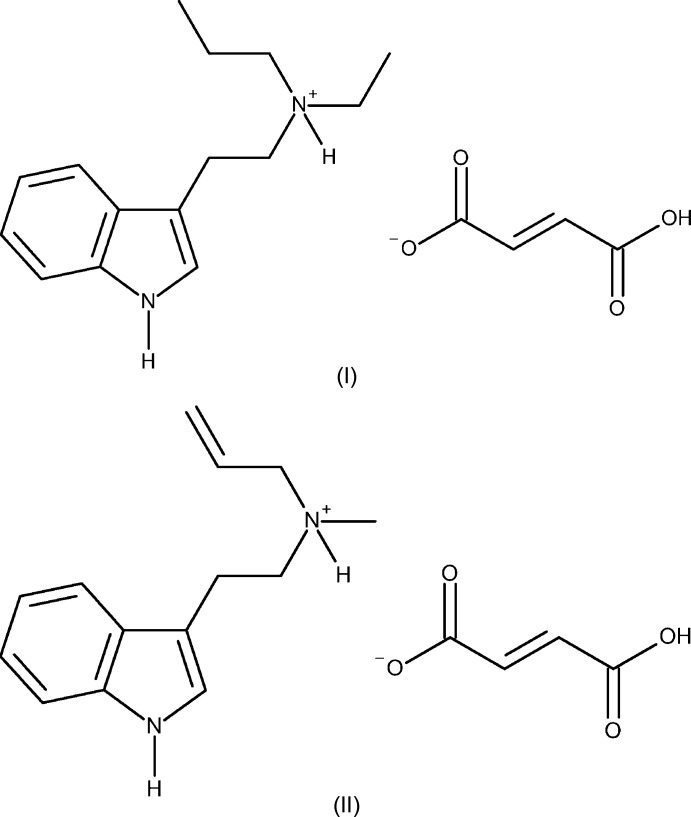


## Structural commentary   

**Figure 1:**
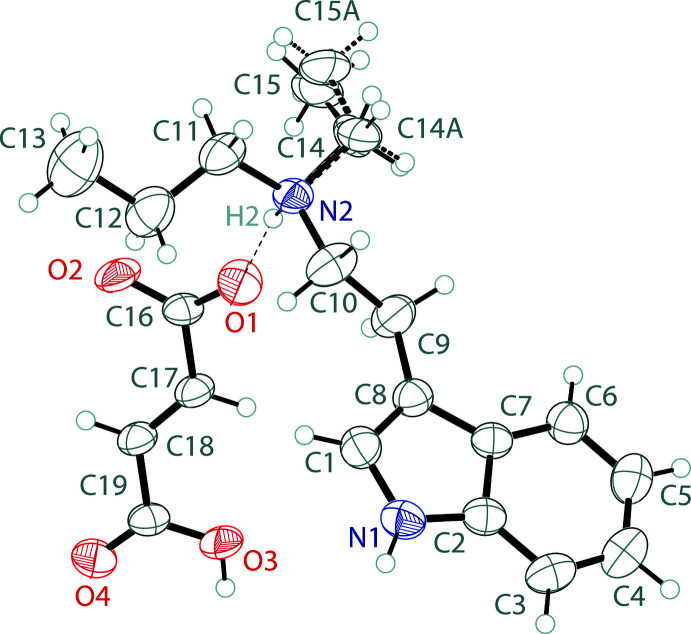
The asymmetric unit of *N*-ethyl-*N*-propyl­tryptammonium hydro­fumarate showing the atom labeling. Displacement ellipsoids are drawn at the 50% probability level. Dashed bonds indicate the disordered component of the structure. The hydrogen bond is shown by a dashed line.

The asymmetric unit of *N*-ethyl-*N*-propyl­tryptammonium hydro­fumarate, (I), contains one tryptammonium cation and one hydro­fumarate anion (Fig. 1 ▸). The cation possesses a near planar indole, with a mean deviation from planarity of 0.008 Å. The ethyl­amino group is slightly turned away from this plane with a C1—C8—C9—C10 torsion angle of 33.9 (4)°. The ethyl group on the *N*-ethyl-*N*-propyl­amine is disordered over two orientations, with a 0.50:0.50 ratio for C14, C15 and C14*A*, C15*A*. The hydro­fumarate anion deviates slightly from planarity with an r.m.s. deviation of 0.135 Å, and a carboxyl­ate to carboxyl­ate plane twist angle of 16.63 (14)°.

**Figure 2:**
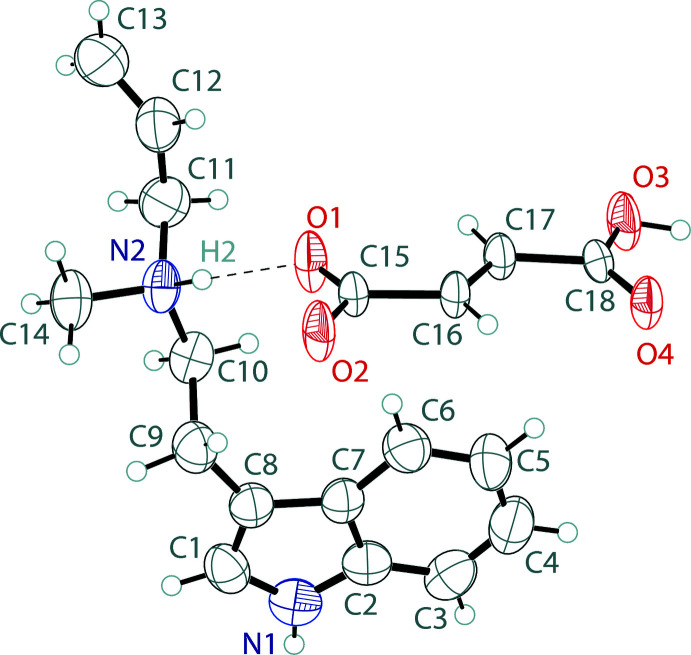
The asymmetric unit of *N*-methyl-*N*-allyl­tryptammonium hydro­fumarate showing the atom labeling. Displacement ellipsoids are drawn at the 50% probability level. The hydrogen bond is shown by a dashed line.

The asymmetric unit of *N*-methyl-*N*-allyl­tryptammonium hydro­fumarate, (II), contains one tryptammonium cation and one hydro­fumarate anion (Fig. 2 ▸). The tryptammonium has a near planar indole, with a mean deviation from planarity of 0.007 Å. The ethyl­amino group is turned away from the plane of the indole, with a C1—C8—C9—C10 torsion angle of −105.5 (5)°. The hydro­fumarate is also near planar, with an r.m.s. deviation of 0.055 Å. The carboxyl­ate is partially delocalized, with C—O distances of 1.239 (5) Å and 1.259 (4) Å.

## Supra­molecular features   

**Figure 3:**
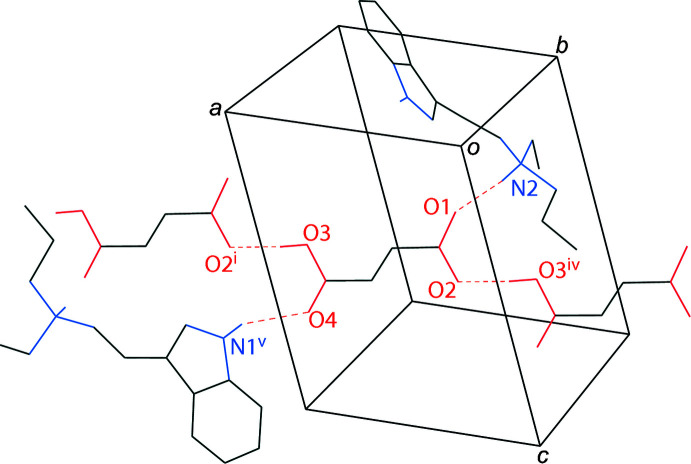
The hydrogen bonding of a hydro­fumarate ion in the structure of *N*-ethyl-*N*-propyl­tryptammonium hydro­fumarate, with hydrogen bonds shown as dashed lines. Only one component of the disorder is shown, and hydrogen atoms not involved in hydrogen bonding are omitted for clarity. Symmetry codes: (i) 1 + *x*, *y*, *z*; (iv) −1 + *x*, *y*, *z*; (v) 2 − *x*, −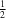 + *y*, 1 − *z*.

The two moieties of the EPT salt, the tryptammonium cation and the hydro­fumarate anion, are held together in the asymmetric unit via N2—H2⋯O1 hydrogen bonds. The indole of another tryptammonium cation inter­acts with a carbonyl oxygen of the hydro­fumarate mol­ecule through an N1—H1⋯O4 hydrogen bond (symmetry operation: 2 − *x*, 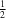 + *y*, 1 − *z*). The hy­droxy group of the hydro­fumarate inter­acts with a carboxyl­ate oxygen of another hydro­fumarate anion through an O3—H3*A*⋯O2 hydrogen bond (symmetry operation: 1 + *x, y*, *z*). The hydro­fumarate anions are linked together in chains along [100], which are linked together by the tryptammonium cations along [010], joining the ions into infinite two-dimensional networks parallel to the (001) plane (Table 1 ▸, Fig. 3 ▸).

**Figure 4:**
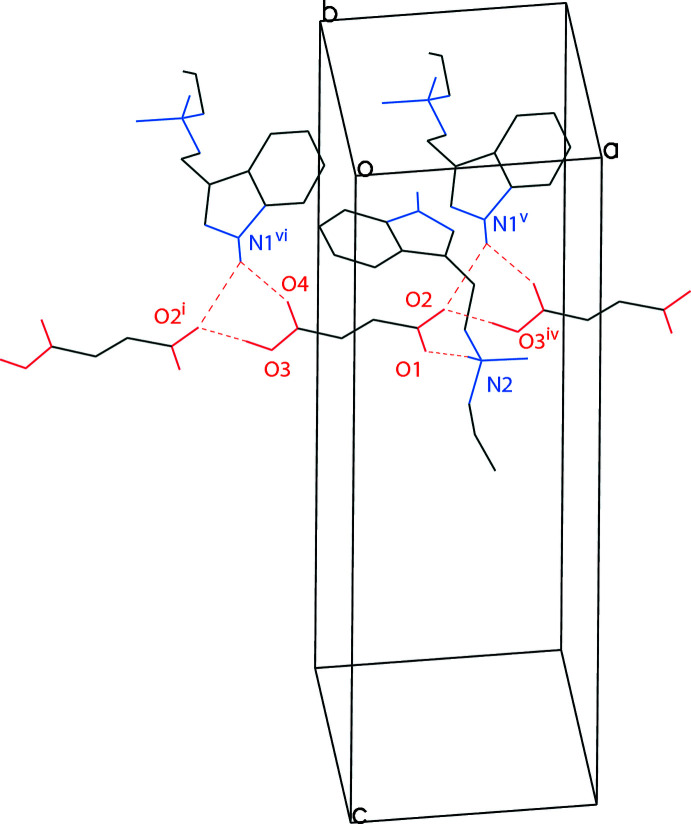
The hydrogen bonding of a hydro­fumarate ion in the structure of *N*-methyl-*N*-allyl­tryptammonium hydro­fumarate, with hydrogen bonds shown as dashed lines. Displacement ellipsoids are drawn at the 50% probability level. Hydrogen atoms not involved in hydrogen bonding are omitted for clarity. Symmetry codes: (i) *x* − 1, *y*, *z*; (iv) 1 + *x*, *y*, *z*; (v) 1 − *x*, 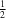 + *y*, 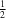 − *z*; (vi) −*x*, 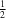 + *y*, 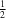 − *z*.

The two moieties of the MALT salt, the tryptammonium cation and the hydro­fumarate anion, are held together in the asymmetric unit *via* N2—H2⋯O1 hydrogen bonds. The hy­droxy group of the hydro­fumarate hydrogen bonds to the carboxyl­ate oxygen of another hydro­fumarate anion through O3—H3*A*⋯O2 hydrogen bonds (symmetry code: −1 + *x*, *y*, *z*). One carbonyl oxygen, O2, of the hydro­fumarate, the indole nitro­gen, N1, of another tryptammonium cation (symmetry code: 1 − *x*, 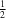 + *y*, 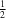 − *z*), and a carbonyl oxygen, O4, of a different hydro­fumarate anion (symmetry code: 1 + *x*, *y*, *z*) combine to form a three-centred (bifurcated) N—H⋯(O,O) hydrogen bond. The hydro­fumarate anions are linked tog­ether in chains along [100], and the tryptammonium cations link these chains together along [010]. The net result in an infinite two-dimensional networks parallel to the (001) plane, similar to what was observed for the EPT (Table 2 ▸, Fig. 4 ▸).

## Database survey   

The compound that is the closest structural comparison to those presented here is *N*-methyl-*N*-iso­propyl­tryptammonium hydro­fumarate (RONSOF: Chadeayne, Pham *et al.*, 2019*a* ▸), which forms similar two-dimensional networks as the two reported compounds, though parallel to (010) instead. The other unsubstituted *N*,*N*-di­alkyl­tryptamines whose structures have been reported are freebase DMT (DMTRYP: Falkenberg, 1972 ▸), the bromide salt of DMT (QQQHIM: Falkenberg, 1972 ▸), and the freebase of *N*-methyl-*N*-propyl­tryptamine (WOHYAW: Chadeayne, Golen & Manke, 2019*b* ▸). The core structure of the tryptamines in these compounds are similar, but the packing is very different given the lack of a similar counter-ion. The reaction of fumaric acid with the freebase of *N*-methyl-*N*-allyl­tryptamine to generate the hydro­fumarate salt is similar to the reactions observed with psilacetin (Nichols & Frescas, 1999 ▸) and norpsilocin (CCDC 1992279: Chadeayne, Pham *et al.*, 2020*b* ▸). The other known tryptammonium fumarate salts are for 4-hy­droxy-*N*-methyl-*N*-iso­propyl­tryptamine (RONSUL: Chadeayne, Pham *et al.*, 2019*a* ▸; CCDC 1987588: Chadeayne, Pham *et al.*, 2020*a* ▸), which join together in infinite parallel chains through N—H⋯O and O—H⋯O hydrogen bonds, 4-acet­oxy-*N*,*N*-di­methyl­tryptamine (HOCJUH: Chadeayne *et al.*, 2019*c* ▸ and XOFDOO: Chadeayne, Golen & Manke, 2019*a* ▸), which joins together in chains through N—H⋯O and O—H⋯O hydrogen bonds, and 4-hy­droxy-*N*,*N*-di­propyl­tryptamine (CCDC 1962339: Chadeayne, Pham *et al.*, 2019*b* ▸), which forms three-dimensional networks through N—H⋯O and O—H⋯O hydrogen bonds. The only other *N*-allyl­tryptamine whose structure has been reported is 5-meth­oxy-*N*,*N*-di­allyl­tryptamine (Chadeayne, Pham *et al.*, 2020*c* ▸), which is reported as the freebase and has not been reported as a salt.

## Synthesis and crystallization   

Single crystals of *N*-ethyl-*N*-propyl­tryptammonium hydro­fumarate suitable for X-ray analysis were obtained from the slow evaporation of an aqueous solution of a commercial sample of EPT fumarate (The Indole Shop).

To prepare *N*-methyl-*N*-allyl­tryptammonium hydro­fumar­ate, 134 mg of a commercial sample of *N*-methyl-*N*-allyl­tryptamine (The Indole Shop), which is a waxy solid that does not crystallize well, were dissolved in 10 mL of methanol, and 68 mg of fumaric acid were added. The mixture was refluxed for 12 h and solvent was removed *in vacuo* to obtain a waxy, yellow product. The material was recrystallized from ethanol to yield colorless single crystals suitable for X-ray diffraction. The product was also characterized by nuclear magnetic resonance. ^1^H NMR (400 MHz, D_2_O): δ 7.69 (*d*, *J* = 7.9 Hz, 1 H, Ar*H*), 7.54 (*d*, *J* = 8.2 Hz, 1 H, Ar*H*), 7.34 (*s*, 1 H, Ar*H*), 7.29 (*t*, *J* = 7.1 Hz, 1 H, Ar*H*), 7.21 (*t*, *J* = 7.1 Hz, 1 H, Ar*H*), 6.66 (*s*, 2 H, C*H*), 5.92–5.82 (*m*, 1 H, C*H*), 5.60–5.56 (*m*, 2 H, C*H*_2_), 3.88–3.83 (*m*, 1 H, C*H*_2_), 3.77–3.72 (*m*, 1 H, C*H*_2_), 3.68–3.57 (*m*, 1 H, C*H*_2_), 3.44–3.37 (*m*, 1 H, C*H*_2_), 3.34–3.21 (*m*, 2 H, C*H*_2_), 2.90 (*s*, 3 H, C*H*_3_). ^13^C NMR (100 MHz, D_2_O): δ 172.2 (*C*OOH), 137.0 (*C*H), 135.5 (Ar*C*), 127.3 (Ar*C*), 126.9 (Ar*C*), 126.2 (Ar*C*), 124.8 (Ar*C*), 122.9 (Ar*C*), 120.1 (Ar*C*), 118.9 (Ar*C*), 112.7 (sp^2^ *C*), 109.0 (sp^2^ *C*), 58.7 (Ak*C*), 55.6 (Ak*C*), 40.1 (Ak*C*), 20.6 (Ak*C*).

## Refinement   

Crystal data, data collection and structure refinement details are summarized in Table 3 ▸. N and O-bound H atoms were located in difference-Fourier maps and refined with distance restraints of N2—C14 = N2—C14*A* =1.50 ± (10), C14—C15 = C14*A*—C15*A* = 1.54 ± (1), N1—H1 = N2—H2 = 0.87± (1) Å for (I) and C12—C13 = 1.400 ± (5), N1—H1 = N2—H2 = 0.87 ± (1), O3—H3*A* 0.88 ± (1) Å for (II). C-bound H atoms were refined as riding with C—H = 0.93–0.97 Å and *U*_iso_(H) = 1.2*U*_eq_(C) or 1.5*U*_eq_(C-meth­yl). The ethyl group of the EPT cation was modeled as a two-component disorder with 50% occupancy for each component for both compounds.

## Supplementary Material

Crystal structure: contains datablock(s) I, II. DOI: 10.1107/S2056989020008683/dx2028sup1.cif

Structure factors: contains datablock(s) I. DOI: 10.1107/S2056989020008683/dx2028Isup2.hkl

Structure factors: contains datablock(s) II. DOI: 10.1107/S2056989020008683/dx2028IIsup3.hkl

CCDC references: 2012495, 2012494

Additional supporting information:  crystallographic information; 3D view; checkCIF report

## Figures and Tables

**Table 1:** Hydrogen-bond geometry (Å, °) for (I)

*D*—H⋯*A*	*D*—H	H⋯*A*	*D*⋯*A*	*D*—H⋯*A*
O3—H3*A*⋯O2^i^	0.87 (4)	1.65 (4)	2.518 (2)	174 (4)
N1—H1⋯O2^ii^	0.88 (1)	2.52 (3)	3.053 (3)	119 (2)
N1—H1⋯O4^iii^	0.88 (1)	2.20 (2)	2.984 (3)	147 (3)
N2—H2⋯O1	0.87 (1)	1.82 (1)	2.682 (2)	169 (2)

Symmetry codes: (i) 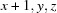; (ii) 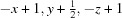; (iii) 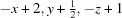.

**Table 2:** Hydrogen-bond geometry (Å, °) for (II)

*D*—H⋯*A*	*D*—H	H⋯*A*	*D*⋯*A*	*D*—H⋯*A*
O3—H3*A*⋯O2^i^	0.88 (1)	1.63 (2)	2.508 (4)	176 (6)
N1—H1⋯O2^ii^	0.87 (1)	2.52 (4)	3.203 (5)	137 (4)
N1—H1⋯O4^iii^	0.87 (1)	2.32 (3)	3.048 (5)	141 (4)
N2—H2⋯O1	0.88 (1)	1.85 (2)	2.695 (4)	163 (4)

Symmetry codes: (i) 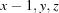; (ii) 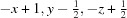; (iii) 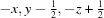.

**Table 3:** Experimental details

	(I)	(II)
Crystal data		
Chemical formula	C_15_H_23_N_2_^+^·C_4_H_3_O_4_^−^	C_14_H_19_N_2_^+^·C_4_H_3_O_4_^−^
*M*_r_	346.42	330.37
Crystal system, space group	Monoclinic, *P*2_1_	Orthorhombic, *P*2_1_2_1_2_1_
Temperature (K)	297	297
*a*, *b*, *c* (Å)	7.4839 (8), 14.1752 (14), 9.6461 (10)	7.9845 (7), 8.5641 (6), 25.649 (2)
α, β, γ (°)	90, 110.537 (3), 90	90, 90, 90
*V* (Å^3^)	958.28 (17)	1753.9 (3)
*Z*	2	4
Radiation type	Mo *K*α	Mo *K*α
μ (mm^−1^)	0.08	0.09
Crystal size (mm)	0.42 × 0.2 × 0.1	0.42 × 0.24 × 0.15

Data collection		
Diffractometer	Bruker D8 Venture CMOS	Bruker D8 Venture CMOS
Absorption correction	Multi-scan (*SADABS*; Bruker, 2018 ▸)	Multi-scan (*SADABS*; Bruker, 2018 ▸)
*T*_min_, *T*_max_	0.703, 0.745	0.681, 0.745
No. of measured, independent and observed [*I* > 2σ(*I*)] reflections	21982, 3570, 3368	49712, 3318, 3036
*R*_int_	0.028	0.046
(sin θ/λ)_max_ (Å^−1^)	0.611	0.610

Refinement		
*R*[*F* ^2^ > 2σ(*F* ^2^)], *wR*(*F* ^2^), *S*	0.034, 0.093, 1.03	0.053, 0.147, 1.10
No. of reflections	3570	3318
No. of parameters	260	228
No. of restraints	7	4
H-atom treatment	H atoms treated by a mixture of independent and constrained refinement	H atoms treated by a mixture of independent and constrained refinement
Δρ_max_, Δρ_min_ (e Å^−3^)	0.13, −0.14	0.25, −0.17
Absolute structure	Flack *x* determined using 1509 quotients [(*I* ^+^)−(*I* ^−^)]/[(*I* ^+^)+(*I* ^−^)] (Parsons *et al.*, 2013 ▸)	Flack *x* determined using 1177 quotients [(*I* ^+^)−(*I* ^−^)]/[(*I* ^+^)+(*I* ^−^)] (Parsons *et al.*, 2013 ▸)
Absolute structure parameter	0.1 (2)	0.0 (3)

Computer programs: *APEX3* and *SAINT* (Bruker, 2018 ▸), *SHELXT2014* (Sheldrick, 2015*a* ▸), *SHELXL2018* (Sheldrick, 2015*b* ▸) and *OLEX2* (Dolomanov *et al.*, 2009 ▸), *publCIF* (Westrip, 2010 ▸).

## supplementary crystallographic information

### [2-(1H-Indol-3-yl)ethyl](methyl)propylazanium 3-carboxyprop-2-enoate (I) Crystal data

**Table:**

C_15_H_23_N_2_^+^·C_4_H_3_O_4_^−^	*F*(000) = 372
*M**_r_* = 346.42	*D*_x_ = 1.201 Mg m^−^^3^
Monoclinic, *P*2_1_	Mo *K*α radiation, λ = 0.71073 Å
*a* = 7.4839 (8) Å	Cell parameters from 9910 reflections
*b* = 14.1752 (14) Å	θ = 2.7–25.7°
*c* = 9.6461 (10) Å	µ = 0.08 mm^−^^1^
β = 110.537 (3)°	*T* = 297 K
*V* = 958.28 (17) Å^3^	PLATE, colourless
*Z* = 2	0.42 × 0.2 × 0.1 mm

### [2-(1H-Indol-3-yl)ethyl](methyl)propylazanium 3-carboxyprop-2-enoate (I) Data collection

**Table:**

Bruker D8 Venture CMOS diffractometer	3368 reflections with *I* > 2σ(*I*)
φ and ω scans	*R*_int_ = 0.028
Absorption correction: multi-scan (SADABS; Bruker, 2018)	θ_max_ = 25.7°, θ_min_ = 2.9°
*T*_min_ = 0.703, *T*_max_ = 0.745	*h* = −9→9
21982 measured reflections	*k* = −16→17
3570 independent reflections	*l* = −11→11

### [2-(1H-Indol-3-yl)ethyl](methyl)propylazanium 3-carboxyprop-2-enoate (I) Refinement

**Table:**

Refinement on *F*^2^	H atoms treated by a mixture of independent and constrained refinement
Least-squares matrix: full	*w* = 1/[σ^2^(*F*_o_^2^) + (0.0553*P*)^2^ + 0.1111*P*] where *P* = (*F*_o_^2^ + 2*F*_c_^2^)/3
*R*[*F*^2^ > 2σ(*F*^2^)] = 0.034	(Δ/σ)_max_ < 0.001
*wR*(*F*^2^) = 0.093	Δρ_max_ = 0.13 e Å^−^^3^
*S* = 1.03	Δρ_min_ = −0.13 e Å^−^^3^
3570 reflections	Extinction correction: SHELXL2018 (Sheldrick 2015b), Fc^*^=kFc[1+0.001xFc^2^λ^3^/sin(2θ)]^-1/4^
260 parameters	Extinction coefficient: 0.10 (3)
7 restraints	Absolute structure: Flack *x* determined using 1509 quotients [(*I*^+^)-(*I*^-^)]/[(*I*^+^)+(*I*^-^)] (Parsons *et al.*, 2013)
Hydrogen site location: mixed	Absolute structure parameter: 0.1 (2)

### [2-(1H-Indol-3-yl)ethyl](methyl)propylazanium 3-carboxyprop-2-enoate (I) Special details

**Table:**

Geometry. All esds (except the esd in the dihedral angle between two l.s. planes) are estimated using the full covariance matrix. The cell esds are taken into account individually in the estimation of esds in distances, angles and torsion angles; correlations between esds in cell parameters are only used when they are defined by crystal symmetry. An approximate (isotropic) treatment of cell esds is used for estimating esds involving l.s. planes.

### [2-(1H-Indol-3-yl)ethyl](methyl)propylazanium 3-carboxyprop-2-enoate (I) Fractional atomic coordinates and isotropic or equivalent isotropic displacement parameters (Å^2^)

**Table:**

	*x*	*y*	*z*	*U*_iso_*/*U*_eq_	Occ. (<1)
O1	0.3041 (2)	0.35379 (16)	0.3731 (2)	0.0636 (6)	
O2	0.3509 (2)	0.30983 (15)	0.60295 (19)	0.0550 (5)	
O3	0.9978 (2)	0.31611 (16)	0.55428 (18)	0.0536 (5)	
H3A	1.120 (5)	0.310 (3)	0.574 (3)	0.072 (9)*	
O4	1.0767 (2)	0.31972 (18)	0.79838 (19)	0.0628 (5)	
C16	0.4059 (3)	0.32758 (15)	0.4973 (2)	0.0374 (5)	
C17	0.6144 (3)	0.31743 (17)	0.5214 (2)	0.0398 (5)	
H17	0.648978	0.306853	0.439068	0.048*	
C18	0.7503 (3)	0.32251 (17)	0.6508 (2)	0.0399 (5)	
H18	0.714909	0.328760	0.733678	0.048*	
C19	0.9582 (3)	0.31898 (16)	0.6750 (2)	0.0391 (5)	
N1	0.5831 (3)	0.69330 (14)	0.1173 (2)	0.0475 (5)	
H1	0.651 (4)	0.7437 (15)	0.155 (3)	0.065 (9)*	
N2	0.0093 (3)	0.45996 (14)	0.2087 (2)	0.0419 (5)	
H2	0.094 (3)	0.4206 (14)	0.265 (2)	0.037 (6)*	
C1	0.4632 (4)	0.64176 (18)	0.1676 (3)	0.0482 (5)	
H1A	0.439846	0.654133	0.254380	0.058*	
C2	0.5831 (3)	0.65482 (16)	−0.0128 (3)	0.0432 (5)	
C3	0.6784 (4)	0.6830 (2)	−0.1055 (3)	0.0564 (6)	
H3	0.758417	0.735307	−0.083577	0.068*	
C4	0.6504 (5)	0.6308 (2)	−0.2307 (3)	0.0652 (8)	
H4	0.711949	0.648402	−0.295338	0.078*	
C5	0.5309 (4)	0.5516 (2)	−0.2637 (3)	0.0608 (7)	
H5	0.516645	0.517150	−0.349024	0.073*	
C6	0.4343 (3)	0.52355 (17)	−0.1731 (3)	0.0486 (6)	
H6	0.354963	0.470958	−0.196039	0.058*	
C7	0.4587 (3)	0.57658 (16)	−0.0449 (2)	0.0402 (5)	
C8	0.3830 (3)	0.56958 (17)	0.0720 (2)	0.0426 (5)	
C9	0.2459 (4)	0.4953 (2)	0.0840 (3)	0.0550 (6)	
H9A	0.316656	0.442561	0.141476	0.066*	
H9B	0.171567	0.472362	−0.014058	0.066*	
C10	0.1139 (4)	0.53459 (19)	0.1570 (3)	0.0558 (6)	
H10A	0.187623	0.572677	0.241152	0.067*	
H10B	0.021466	0.575561	0.087367	0.067*	
C11	−0.0973 (4)	0.5018 (2)	0.2997 (3)	0.0533 (6)	
H11A	−0.187131	0.548153	0.240391	0.064*	
H11B	−0.169895	0.452430	0.325308	0.064*	
C12	0.0298 (5)	0.5481 (2)	0.4390 (4)	0.0681 (8)	
H12A	0.131071	0.504882	0.492510	0.082*	
H12B	0.087974	0.603660	0.414204	0.082*	
C13	−0.0794 (7)	0.5757 (3)	0.5350 (5)	0.0955 (12)	
H13A	0.005795	0.602901	0.625092	0.143*	
H13B	−0.139369	0.520900	0.557867	0.143*	
H13C	−0.175280	0.621019	0.484177	0.143*	
C14	−0.108 (2)	0.3898 (9)	0.0926 (13)	0.052 (3)	0.5
H14A	−0.039029	0.375936	0.026374	0.062*	0.5
H14B	−0.227066	0.419643	0.034355	0.062*	0.5
C15	−0.1542 (12)	0.2941 (8)	0.1545 (13)	0.080 (3)	0.5
H15A	−0.038462	0.266848	0.220982	0.120*	0.5
H15B	−0.212182	0.251470	0.073842	0.120*	0.5
H15C	−0.240528	0.305477	0.206447	0.120*	0.5
C14A	−0.126 (2)	0.4138 (10)	0.0749 (14)	0.058 (4)	0.5
H14C	−0.057294	0.389263	0.014130	0.070*	0.5
H14D	−0.218490	0.459560	0.016775	0.070*	0.5
C15A	−0.2296 (15)	0.3328 (8)	0.1217 (12)	0.087 (3)	0.5
H15D	−0.137282	0.289846	0.184512	0.131*	0.5
H15E	−0.309931	0.299929	0.035253	0.131*	0.5
H15F	−0.306261	0.358052	0.174491	0.131*	0.5

### [2-(1H-Indol-3-yl)ethyl](methyl)propylazanium 3-carboxyprop-2-enoate (I) Atomic displacement parameters (Å^2^)

**Table:**

	*U*^11^	*U*^22^	*U*^33^	*U*^12^	*U*^13^	*U*^23^
O1	0.0410 (9)	0.0958 (15)	0.0500 (10)	0.0262 (9)	0.0110 (8)	0.0180 (10)
O2	0.0258 (7)	0.0854 (12)	0.0572 (9)	0.0073 (8)	0.0187 (7)	0.0215 (9)
O3	0.0252 (7)	0.0864 (12)	0.0511 (9)	0.0040 (8)	0.0157 (7)	0.0065 (9)
O4	0.0311 (8)	0.1007 (15)	0.0512 (10)	0.0082 (9)	0.0076 (7)	0.0015 (10)
C16	0.0251 (9)	0.0410 (11)	0.0451 (11)	0.0050 (8)	0.0112 (8)	0.0038 (9)
C17	0.0281 (9)	0.0503 (12)	0.0440 (11)	0.0047 (9)	0.0163 (8)	0.0069 (10)
C18	0.0284 (9)	0.0497 (11)	0.0445 (10)	0.0041 (9)	0.0162 (8)	0.0038 (10)
C19	0.0276 (9)	0.0425 (11)	0.0472 (11)	0.0026 (9)	0.0131 (9)	0.0034 (9)
N1	0.0426 (10)	0.0434 (11)	0.0539 (12)	−0.0040 (8)	0.0136 (9)	−0.0061 (9)
N2	0.0284 (8)	0.0526 (11)	0.0423 (10)	0.0059 (8)	0.0094 (7)	0.0081 (8)
C1	0.0457 (13)	0.0521 (13)	0.0465 (12)	0.0046 (10)	0.0160 (10)	−0.0024 (11)
C2	0.0353 (11)	0.0415 (12)	0.0498 (12)	0.0047 (8)	0.0111 (10)	0.0041 (9)
C3	0.0500 (13)	0.0529 (14)	0.0715 (17)	−0.0045 (11)	0.0279 (12)	0.0084 (13)
C4	0.0723 (19)	0.0686 (18)	0.0679 (17)	0.0023 (15)	0.0410 (16)	0.0081 (15)
C5	0.0687 (17)	0.0668 (17)	0.0536 (15)	0.0047 (13)	0.0297 (13)	−0.0029 (12)
C6	0.0501 (13)	0.0469 (14)	0.0487 (12)	−0.0002 (10)	0.0172 (10)	−0.0036 (10)
C7	0.0331 (11)	0.0407 (11)	0.0450 (12)	0.0032 (8)	0.0115 (9)	0.0019 (9)
C8	0.0388 (11)	0.0446 (12)	0.0450 (12)	0.0005 (9)	0.0156 (9)	−0.0011 (9)
C9	0.0587 (15)	0.0547 (14)	0.0618 (15)	−0.0092 (12)	0.0339 (13)	−0.0066 (12)
C10	0.0507 (13)	0.0509 (14)	0.0748 (17)	0.0091 (11)	0.0332 (12)	0.0182 (13)
C11	0.0426 (12)	0.0606 (15)	0.0620 (15)	0.0133 (11)	0.0250 (12)	0.0104 (12)
C12	0.076 (2)	0.0633 (18)	0.0769 (19)	−0.0019 (14)	0.0421 (16)	−0.0098 (14)
C13	0.123 (3)	0.081 (2)	0.106 (3)	0.008 (2)	0.069 (3)	−0.011 (2)
C14	0.038 (4)	0.074 (6)	0.033 (4)	0.009 (4)	−0.001 (3)	0.000 (4)
C15	0.046 (4)	0.110 (8)	0.085 (6)	−0.027 (4)	0.023 (4)	−0.055 (5)
C14A	0.031 (5)	0.090 (8)	0.048 (5)	−0.009 (6)	0.006 (3)	0.008 (4)
C15A	0.074 (6)	0.115 (9)	0.085 (6)	−0.062 (6)	0.042 (6)	−0.040 (6)

### [2-(1H-Indol-3-yl)ethyl](methyl)propylazanium 3-carboxyprop-2-enoate (I) Geometric parameters (Å, º)

**Table:**

O1—C16	1.230 (3)	C6—C7	1.403 (3)
O2—C16	1.251 (3)	C7—C8	1.432 (3)
O3—H3A	0.87 (4)	C8—C9	1.503 (3)
O3—C19	1.299 (3)	C9—H9A	0.9700
O4—C19	1.210 (3)	C9—H9B	0.9700
C16—C17	1.502 (3)	C9—C10	1.506 (4)
C17—H17	0.9300	C10—H10A	0.9700
C17—C18	1.307 (3)	C10—H10B	0.9700
C18—H18	0.9300	C11—H11A	0.9700
C18—C19	1.491 (3)	C11—H11B	0.9700
N1—H1	0.880 (14)	C11—C12	1.498 (4)
N1—C1	1.371 (3)	C12—H12A	0.9700
N1—C2	1.368 (3)	C12—H12B	0.9700
N2—H2	0.873 (13)	C12—C13	1.487 (4)
N2—C10	1.502 (3)	C13—H13A	0.9600
N2—C11	1.500 (3)	C13—H13B	0.9600
N2—C14	1.524 (10)	C13—H13C	0.9600
N2—C14A	1.485 (10)	C14—H14A	0.9700
C1—H1A	0.9300	C14—H14B	0.9700
C1—C8	1.367 (3)	C14—C15	1.569 (11)
C2—C3	1.384 (3)	C15—H15A	0.9600
C2—C7	1.411 (3)	C15—H15B	0.9600
C3—H3	0.9300	C15—H15C	0.9600
C3—C4	1.367 (4)	C14A—H14C	0.9700
C4—H4	0.9300	C14A—H14D	0.9700
C4—C5	1.401 (4)	C14A—C15A	1.539 (11)
C5—H5	0.9300	C15A—H15D	0.9600
C5—C6	1.375 (4)	C15A—H15E	0.9600
C6—H6	0.9300	C15A—H15F	0.9600
			
C19—O3—H3A	111 (2)	H9A—C9—H9B	108.1
O1—C16—O2	125.89 (18)	C10—C9—H9A	109.5
O1—C16—C17	115.76 (18)	C10—C9—H9B	109.5
O2—C16—C17	118.35 (17)	N2—C10—C9	113.5 (2)
C16—C17—H17	117.9	N2—C10—H10A	108.9
C18—C17—C16	124.14 (19)	N2—C10—H10B	108.9
C18—C17—H17	117.9	C9—C10—H10A	108.9
C17—C18—H18	117.7	C9—C10—H10B	108.9
C17—C18—C19	124.55 (19)	H10A—C10—H10B	107.7
C19—C18—H18	117.7	N2—C11—H11A	108.9
O3—C19—C18	114.52 (18)	N2—C11—H11B	108.9
O4—C19—O3	124.26 (18)	H11A—C11—H11B	107.7
O4—C19—C18	121.21 (19)	C12—C11—N2	113.4 (2)
C1—N1—H1	130 (2)	C12—C11—H11A	108.9
C2—N1—H1	121 (2)	C12—C11—H11B	108.9
C2—N1—C1	108.84 (19)	C11—C12—H12A	109.4
C10—N2—H2	107.8 (16)	C11—C12—H12B	109.4
C10—N2—C14	116.6 (6)	H12A—C12—H12B	108.0
C11—N2—H2	108.1 (16)	C13—C12—C11	111.1 (3)
C11—N2—C10	111.2 (2)	C13—C12—H12A	109.4
C11—N2—C14	113.7 (7)	C13—C12—H12B	109.4
C14—N2—H2	98.1 (17)	C12—C13—H13A	109.5
C14A—N2—H2	112.4 (17)	C12—C13—H13B	109.5
C14A—N2—C10	107.3 (5)	C12—C13—H13C	109.5
C14A—N2—C11	110.0 (7)	H13A—C13—H13B	109.5
N1—C1—H1A	124.8	H13A—C13—H13C	109.5
C8—C1—N1	110.3 (2)	H13B—C13—H13C	109.5
C8—C1—H1A	124.8	N2—C14—H14A	108.4
N1—C2—C3	130.1 (2)	N2—C14—H14B	108.4
N1—C2—C7	107.6 (2)	N2—C14—C15	115.6 (8)
C3—C2—C7	122.3 (2)	H14A—C14—H14B	107.5
C2—C3—H3	121.3	C15—C14—H14A	108.4
C4—C3—C2	117.4 (3)	C15—C14—H14B	108.4
C4—C3—H3	121.3	C14—C15—H15A	109.5
C3—C4—H4	119.3	C14—C15—H15B	109.5
C3—C4—C5	121.5 (3)	C14—C15—H15C	109.5
C5—C4—H4	119.3	H15A—C15—H15B	109.5
C4—C5—H5	119.2	H15A—C15—H15C	109.5
C6—C5—C4	121.5 (3)	H15B—C15—H15C	109.5
C6—C5—H5	119.2	N2—C14A—H14C	109.8
C5—C6—H6	121.0	N2—C14A—H14D	109.8
C5—C6—C7	118.1 (2)	N2—C14A—C15A	109.5 (9)
C7—C6—H6	121.0	H14C—C14A—H14D	108.2
C2—C7—C8	107.13 (19)	C15A—C14A—H14C	109.8
C6—C7—C2	119.1 (2)	C15A—C14A—H14D	109.8
C6—C7—C8	133.8 (2)	C14A—C15A—H15D	109.5
C1—C8—C7	106.1 (2)	C14A—C15A—H15E	109.5
C1—C8—C9	128.5 (2)	C14A—C15A—H15F	109.5
C7—C8—C9	125.4 (2)	H15D—C15A—H15E	109.5
C8—C9—H9A	109.5	H15D—C15A—H15F	109.5
C8—C9—H9B	109.5	H15E—C15A—H15F	109.5
C8—C9—C10	110.8 (2)		
			
O1—C16—C17—C18	155.5 (3)	C3—C4—C5—C6	1.0 (5)
O2—C16—C17—C18	−24.0 (4)	C4—C5—C6—C7	−0.2 (4)
C16—C17—C18—C19	−175.7 (2)	C5—C6—C7—C2	−1.2 (3)
C17—C18—C19—O3	4.1 (3)	C5—C6—C7—C8	179.5 (2)
C17—C18—C19—O4	−176.6 (3)	C6—C7—C8—C1	179.2 (2)
N1—C1—C8—C7	0.3 (3)	C6—C7—C8—C9	0.2 (4)
N1—C1—C8—C9	179.2 (2)	C7—C2—C3—C4	−1.0 (4)
N1—C2—C3—C4	−179.5 (3)	C7—C8—C9—C10	−147.4 (2)
N1—C2—C7—C6	−179.4 (2)	C8—C9—C10—N2	−164.8 (2)
N1—C2—C7—C8	0.1 (2)	C10—N2—C11—C12	−62.5 (3)
N2—C11—C12—C13	−171.7 (3)	C10—N2—C14—C15	159.0 (9)
C1—N1—C2—C3	178.7 (2)	C10—N2—C14A—C15A	176.9 (9)
C1—N1—C2—C7	0.1 (2)	C11—N2—C10—C9	171.7 (2)
C1—C8—C9—C10	33.9 (4)	C11—N2—C14—C15	−69.5 (13)
C2—N1—C1—C8	−0.2 (3)	C11—N2—C14A—C15A	−61.9 (12)
C2—C3—C4—C5	−0.4 (4)	C14—N2—C10—C9	−55.8 (8)
C2—C7—C8—C1	−0.2 (2)	C14—N2—C11—C12	163.5 (6)
C2—C7—C8—C9	−179.2 (2)	C14A—N2—C10—C9	−68.0 (8)
C3—C2—C7—C6	1.8 (3)	C14A—N2—C11—C12	178.7 (6)
C3—C2—C7—C8	−178.7 (2)		

### [2-(1H-Indol-3-yl)ethyl](methyl)propylazanium 3-carboxyprop-2-enoate (I) Hydrogen-bond geometry (Å, º)

**Table:**

*D*—H···*A*	*D*—H	H···*A*	*D*···*A*	*D*—H···*A*
O3—H3*A*···O2^i^	0.87 (4)	1.65 (4)	2.518 (2)	174 (4)
N1—H1···O2^ii^	0.88 (1)	2.52 (3)	3.053 (3)	119 (2)
N1—H1···O4^iii^	0.88 (1)	2.20 (2)	2.984 (3)	147 (3)
N2—H2···O1	0.87 (1)	1.82 (1)	2.682 (2)	169 (2)

Symmetry codes: (i) *x*+1, *y*, *z*; (ii) −*x*+1, *y*+1/2, −*z*+1; (iii) −*x*+2, *y*+1/2, −*z*+1.

### [2-(1H-Indol-3-yl)ethyl](methyl)(prop-2-en-1-yl)azanium 3-carboxyprop-2-enoate (II) Crystal data

**Table:**

C_14_H_19_N_2_^+^·C_4_H_3_O_4_^−^	*D*_x_ = 1.251 Mg m^−^^3^
*M**_r_* = 330.37	Mo *K*α radiation, λ = 0.71073 Å
Orthorhombic, *P*2_1_2_1_2_1_	Cell parameters from 9827 reflections
*a* = 7.9845 (7) Å	θ = 2.7–25.6°
*b* = 8.5641 (6) Å	µ = 0.09 mm^−^^1^
*c* = 25.649 (2) Å	*T* = 297 K
*V* = 1753.9 (3) Å^3^	BLOCK, colourless
*Z* = 4	0.42 × 0.24 × 0.15 mm
*F*(000) = 704	

### [2-(1H-Indol-3-yl)ethyl](methyl)(prop-2-en-1-yl)azanium 3-carboxyprop-2-enoate (II) Data collection

**Table:**

Bruker D8 Venture CMOS diffractometer	3036 reflections with *I* > 2σ(*I*)
φ and ω scans	*R*_int_ = 0.046
Absorption correction: multi-scan (SADABS; Bruker, 2018)	θ_max_ = 25.7°, θ_min_ = 2.7°
*T*_min_ = 0.681, *T*_max_ = 0.745	*h* = −9→9
49712 measured reflections	*k* = −10→10
3318 independent reflections	*l* = −31→31

### [2-(1H-Indol-3-yl)ethyl](methyl)(prop-2-en-1-yl)azanium 3-carboxyprop-2-enoate (II) Refinement

**Table:**

Refinement on *F*^2^	H atoms treated by a mixture of independent and constrained refinement
Least-squares matrix: full	*w* = 1/[σ^2^(*F*_o_^2^) + (0.0615*P*)^2^ + 0.9658*P*] where *P* = (*F*_o_^2^ + 2*F*_c_^2^)/3
*R*[*F*^2^ > 2σ(*F*^2^)] = 0.053	(Δ/σ)_max_ < 0.001
*wR*(*F*^2^) = 0.147	Δρ_max_ = 0.25 e Å^−^^3^
*S* = 1.10	Δρ_min_ = −0.17 e Å^−^^3^
3318 reflections	Extinction correction: SHELXL2018 (Sheldrick, 2015b), Fc^*^=kFc[1+0.001xFc^2^λ^3^/sin(2θ)]^-1/4^
228 parameters	Extinction coefficient: 0.035 (7)
4 restraints	Absolute structure: Flack *x* determined using 1177 quotients [(*I*^+^)-(*I*^-^)]/[(*I*^+^)+(*I*^-^)] (Parsons *et al.*, 2013)
Hydrogen site location: mixed	Absolute structure parameter: 0.0 (3)

### [2-(1H-Indol-3-yl)ethyl](methyl)(prop-2-en-1-yl)azanium 3-carboxyprop-2-enoate (II) Special details

**Table:**

Geometry. All esds (except the esd in the dihedral angle between two l.s. planes) are estimated using the full covariance matrix. The cell esds are taken into account individually in the estimation of esds in distances, angles and torsion angles; correlations between esds in cell parameters are only used when they are defined by crystal symmetry. An approximate (isotropic) treatment of cell esds is used for estimating esds involving l.s. planes.

### [2-(1H-Indol-3-yl)ethyl](methyl)(prop-2-en-1-yl)azanium 3-carboxyprop-2-enoate (II) Fractional atomic coordinates and isotropic or equivalent isotropic displacement parameters (Å^2^)

**Table:**

	*x*	*y*	*z*	*U*_iso_*/*U*_eq_	
O1	0.3826 (3)	0.6187 (3)	0.43011 (14)	0.0593 (8)	
O2	0.4928 (3)	0.8559 (3)	0.42479 (15)	0.0601 (9)	
O3	−0.2263 (3)	0.7304 (3)	0.43758 (14)	0.0586 (8)	
H3A	−0.323 (4)	0.779 (6)	0.433 (2)	0.088*	
O4	−0.1284 (3)	0.9688 (3)	0.42243 (12)	0.0489 (7)	
N1	0.3281 (5)	0.4814 (4)	0.17877 (14)	0.0575 (9)	
H1	0.321 (6)	0.465 (6)	0.1455 (7)	0.069*	
N2	0.5754 (4)	0.3846 (4)	0.39368 (14)	0.0491 (8)	
H2	0.532 (5)	0.470 (3)	0.4071 (16)	0.059*	
C1	0.4682 (6)	0.4583 (5)	0.20814 (18)	0.0567 (10)	
H1A	0.567191	0.414312	0.195924	0.068*	
C2	0.2087 (6)	0.5503 (5)	0.20900 (16)	0.0503 (10)	
C3	0.0462 (6)	0.5976 (5)	0.19700 (19)	0.0618 (12)	
H3	0.002055	0.584043	0.163752	0.074*	
C4	−0.0465 (7)	0.6653 (6)	0.2362 (2)	0.0716 (14)	
H4	−0.154783	0.699179	0.229132	0.086*	
C5	0.0179 (7)	0.6841 (6)	0.2863 (2)	0.0680 (13)	
H5	−0.047868	0.730535	0.311898	0.082*	
C6	0.1763 (6)	0.6355 (5)	0.29841 (18)	0.0587 (11)	
H6	0.217240	0.646538	0.332154	0.070*	
C7	0.2761 (5)	0.5689 (4)	0.25947 (15)	0.0463 (9)	
C8	0.4428 (5)	0.5086 (5)	0.25791 (16)	0.0497 (9)	
C9	0.5600 (6)	0.4988 (6)	0.30303 (18)	0.0603 (11)	
H9A	0.563040	0.598416	0.321004	0.072*	
H9B	0.672096	0.475965	0.290581	0.072*	
C10	0.5038 (6)	0.3709 (5)	0.34088 (17)	0.0544 (10)	
H10A	0.382653	0.373599	0.343539	0.065*	
H10B	0.534845	0.270232	0.326513	0.065*	
C11	0.5071 (7)	0.2583 (5)	0.42859 (19)	0.0636 (12)	
H11A	0.387600	0.248536	0.422582	0.076*	
H11B	0.558853	0.159744	0.419276	0.076*	
C12	0.5358 (6)	0.2881 (6)	0.48389 (18)	0.0646 (12)	
H12	0.506357	0.384831	0.497556	0.077*	
C13	0.6037 (7)	0.1801 (7)	0.5159 (2)	0.0822 (16)	
H13A	0.633969	0.082723	0.502960	0.099*	
H13B	0.620232	0.203175	0.551004	0.099*	
C14	0.7626 (5)	0.3814 (6)	0.3950 (2)	0.0652 (12)	
H14A	0.800416	0.394052	0.430234	0.098*	
H14B	0.805793	0.464805	0.373889	0.098*	
H14C	0.801662	0.283251	0.381584	0.098*	
C15	0.3711 (4)	0.7629 (4)	0.42824 (14)	0.0380 (8)	
C16	0.2018 (4)	0.8376 (4)	0.42840 (15)	0.0397 (8)	
H16	0.195401	0.945177	0.423952	0.048*	
C17	0.0620 (4)	0.7596 (4)	0.43446 (15)	0.0419 (8)	
H17	0.068725	0.652892	0.440823	0.050*	
C18	−0.1070 (4)	0.8323 (4)	0.43174 (14)	0.0367 (8)	

### [2-(1H-Indol-3-yl)ethyl](methyl)(prop-2-en-1-yl)azanium 3-carboxyprop-2-enoate (II) Atomic displacement parameters (Å^2^)

**Table:**

	*U*^11^	*U*^22^	*U*^33^	*U*^12^	*U*^13^	*U*^23^
O1	0.0322 (14)	0.0410 (15)	0.105 (2)	0.0101 (11)	0.0061 (15)	−0.0037 (16)
O2	0.0208 (13)	0.0509 (16)	0.109 (2)	0.0011 (11)	0.0006 (14)	0.0053 (16)
O3	0.0214 (13)	0.0513 (16)	0.103 (2)	−0.0010 (11)	0.0052 (14)	0.0062 (16)
O4	0.0249 (12)	0.0432 (14)	0.0785 (19)	0.0046 (10)	−0.0010 (12)	0.0031 (13)
N1	0.063 (2)	0.054 (2)	0.055 (2)	−0.0092 (19)	0.0009 (18)	−0.0011 (17)
N2	0.0420 (18)	0.0395 (17)	0.066 (2)	0.0072 (14)	−0.0031 (16)	−0.0080 (15)
C1	0.050 (2)	0.049 (2)	0.071 (3)	−0.0025 (19)	0.013 (2)	0.001 (2)
C2	0.056 (2)	0.040 (2)	0.055 (2)	−0.0089 (18)	0.001 (2)	0.0034 (17)
C3	0.059 (3)	0.059 (3)	0.067 (3)	−0.011 (2)	−0.014 (2)	0.010 (2)
C4	0.054 (3)	0.067 (3)	0.094 (4)	0.009 (2)	−0.006 (3)	0.012 (3)
C5	0.059 (3)	0.065 (3)	0.080 (3)	0.020 (2)	0.007 (2)	−0.003 (2)
C6	0.061 (3)	0.058 (3)	0.058 (2)	0.002 (2)	0.002 (2)	−0.004 (2)
C7	0.046 (2)	0.0356 (18)	0.057 (2)	−0.0016 (16)	0.0011 (18)	0.0038 (16)
C8	0.049 (2)	0.044 (2)	0.056 (2)	−0.0004 (19)	0.0021 (18)	0.0045 (17)
C9	0.046 (2)	0.064 (3)	0.070 (3)	−0.001 (2)	0.001 (2)	0.003 (2)
C10	0.050 (2)	0.049 (2)	0.064 (3)	0.0029 (19)	−0.0073 (19)	−0.0018 (18)
C11	0.071 (3)	0.051 (2)	0.068 (3)	0.003 (2)	0.003 (2)	0.001 (2)
C12	0.056 (3)	0.068 (3)	0.070 (3)	0.011 (2)	0.001 (2)	−0.006 (2)
C13	0.062 (3)	0.101 (4)	0.083 (4)	−0.004 (3)	0.007 (3)	0.020 (3)
C14	0.041 (2)	0.071 (3)	0.084 (3)	0.005 (2)	−0.008 (2)	−0.009 (3)
C15	0.0235 (16)	0.0409 (18)	0.050 (2)	0.0058 (14)	0.0009 (15)	−0.0017 (16)
C16	0.0231 (16)	0.0400 (18)	0.056 (2)	0.0058 (13)	−0.0015 (15)	−0.0002 (16)
C17	0.0247 (16)	0.0411 (17)	0.060 (2)	0.0039 (14)	0.0020 (15)	0.0024 (16)
C18	0.0203 (15)	0.0443 (19)	0.0455 (18)	0.0015 (13)	0.0026 (14)	−0.0019 (16)

### [2-(1H-Indol-3-yl)ethyl](methyl)(prop-2-en-1-yl)azanium 3-carboxyprop-2-enoate (II) Geometric parameters (Å, º)

**Table:**

O1—C15	1.239 (5)	C6—C7	1.399 (6)
O2—C15	1.259 (4)	C7—C8	1.428 (6)
O3—H3A	0.884 (14)	C8—C9	1.491 (6)
O3—C18	1.301 (4)	C9—H9A	0.9700
O4—C18	1.205 (4)	C9—H9B	0.9700
N1—H1	0.868 (13)	C9—C10	1.531 (6)
N1—C1	1.363 (6)	C10—H10A	0.9700
N1—C2	1.363 (6)	C10—H10B	0.9700
N2—H2	0.877 (13)	C11—H11A	0.9700
N2—C10	1.475 (5)	C11—H11B	0.9700
N2—C11	1.506 (6)	C11—C12	1.459 (7)
N2—C14	1.495 (5)	C12—H12	0.9300
C1—H1A	0.9300	C12—C13	1.351 (5)
C1—C8	1.362 (6)	C13—H13A	0.9300
C2—C3	1.394 (6)	C13—H13B	0.9300
C2—C7	1.411 (6)	C14—H14A	0.9600
C3—H3	0.9300	C14—H14B	0.9600
C3—C4	1.377 (8)	C14—H14C	0.9600
C4—H4	0.9300	C15—C16	1.495 (4)
C4—C5	1.393 (7)	C16—H16	0.9300
C5—H5	0.9300	C16—C17	1.310 (5)
C5—C6	1.367 (7)	C17—H17	0.9300
C6—H6	0.9300	C17—C18	1.488 (5)
			
C18—O3—H3A	108 (4)	C10—C9—H9A	109.6
C1—N1—H1	125 (3)	C10—C9—H9B	109.6
C2—N1—H1	126 (3)	N2—C10—C9	114.3 (4)
C2—N1—C1	108.8 (4)	N2—C10—H10A	108.7
C10—N2—H2	106 (3)	N2—C10—H10B	108.7
C10—N2—C11	110.4 (3)	C9—C10—H10A	108.7
C10—N2—C14	114.0 (4)	C9—C10—H10B	108.7
C11—N2—H2	103 (3)	H10A—C10—H10B	107.6
C14—N2—H2	114 (3)	N2—C11—H11A	108.9
C14—N2—C11	109.6 (4)	N2—C11—H11B	108.9
N1—C1—H1A	124.8	H11A—C11—H11B	107.7
C8—C1—N1	110.5 (4)	C12—C11—N2	113.3 (4)
C8—C1—H1A	124.8	C12—C11—H11A	108.9
N1—C2—C3	130.6 (4)	C12—C11—H11B	108.9
N1—C2—C7	107.7 (4)	C11—C12—H12	118.8
C3—C2—C7	121.7 (4)	C13—C12—C11	122.4 (5)
C2—C3—H3	121.3	C13—C12—H12	118.8
C4—C3—C2	117.5 (4)	C12—C13—H13A	120.0
C4—C3—H3	121.3	C12—C13—H13B	120.0
C3—C4—H4	119.2	H13A—C13—H13B	120.0
C3—C4—C5	121.6 (5)	N2—C14—H14A	109.5
C5—C4—H4	119.2	N2—C14—H14B	109.5
C4—C5—H5	119.5	N2—C14—H14C	109.5
C6—C5—C4	121.1 (5)	H14A—C14—H14B	109.5
C6—C5—H5	119.5	H14A—C14—H14C	109.5
C5—C6—H6	120.4	H14B—C14—H14C	109.5
C5—C6—C7	119.3 (4)	O1—C15—O2	125.1 (3)
C7—C6—H6	120.4	O1—C15—C16	119.5 (3)
C2—C7—C8	106.8 (4)	O2—C15—C16	115.3 (3)
C6—C7—C2	118.9 (4)	C15—C16—H16	118.2
C6—C7—C8	134.3 (4)	C17—C16—C15	123.6 (3)
C1—C8—C7	106.2 (4)	C17—C16—H16	118.2
C1—C8—C9	128.0 (4)	C16—C17—H17	118.2
C7—C8—C9	125.7 (4)	C16—C17—C18	123.6 (3)
C8—C9—H9A	109.6	C18—C17—H17	118.2
C8—C9—H9B	109.6	O3—C18—C17	112.2 (3)
C8—C9—C10	110.4 (4)	O4—C18—O3	124.7 (3)
H9A—C9—H9B	108.1	O4—C18—C17	122.9 (3)
			
O1—C15—C16—C17	5.5 (6)	C3—C4—C5—C6	−0.2 (8)
O2—C15—C16—C17	−176.3 (4)	C4—C5—C6—C7	1.4 (7)
N1—C1—C8—C7	−0.7 (5)	C5—C6—C7—C2	−1.5 (6)
N1—C1—C8—C9	177.1 (4)	C5—C6—C7—C8	179.1 (5)
N1—C2—C3—C4	−179.9 (4)	C6—C7—C8—C1	179.5 (4)
N1—C2—C7—C6	−179.1 (4)	C6—C7—C8—C9	1.7 (7)
N1—C2—C7—C8	0.4 (4)	C7—C2—C3—C4	0.7 (6)
N2—C11—C12—C13	−129.7 (5)	C7—C8—C9—C10	71.9 (5)
C1—N1—C2—C3	179.6 (4)	C8—C9—C10—N2	−161.2 (4)
C1—N1—C2—C7	−0.9 (5)	C10—N2—C11—C12	−164.5 (4)
C1—C8—C9—C10	−105.5 (5)	C11—N2—C10—C9	177.5 (4)
C2—N1—C1—C8	1.0 (5)	C14—N2—C10—C9	−58.6 (5)
C2—C3—C4—C5	−0.9 (7)	C14—N2—C11—C12	69.1 (5)
C2—C7—C8—C1	0.2 (4)	C15—C16—C17—C18	−176.5 (4)
C2—C7—C8—C9	−177.7 (4)	C16—C17—C18—O3	178.2 (4)
C3—C2—C7—C6	0.5 (6)	C16—C17—C18—O4	1.9 (6)
C3—C2—C7—C8	180.0 (4)		

### [2-(1H-Indol-3-yl)ethyl](methyl)(prop-2-en-1-yl)azanium 3-carboxyprop-2-enoate (II) Hydrogen-bond geometry (Å, º)

**Table:**

*D*—H···*A*	*D*—H	H···*A*	*D*···*A*	*D*—H···*A*
O3—H3*A*···O2^i^	0.88 (1)	1.63 (2)	2.508 (4)	176 (6)
N1—H1···O2^ii^	0.87 (1)	2.52 (4)	3.203 (5)	137 (4)
N1—H1···O4^iii^	0.87 (1)	2.32 (3)	3.048 (5)	141 (4)
N2—H2···O1	0.88 (1)	1.85 (2)	2.695 (4)	163 (4)

Symmetry codes: (i) *x*−1, *y*, *z*; (ii) −*x*+1, *y*−1/2, −*z*+1/2; (iii) −*x*, *y*−1/2, −*z*+1/2.
